# Regional disease in head and neck cutaneous squamous cell carcinoma: the role of primary tumor characteristics and number of nodal metastases

**DOI:** 10.1007/s00405-021-06944-w

**Published:** 2021-06-25

**Authors:** Alberto Grammatica, Michele Tomasoni, Milena Fior, Emanuela Ulaj, Tommaso Gualtieri, Paolo Bossi, Simonetta Battocchio, Davide Lombardi, Alberto Deganello, Davide Mattavelli, Piero Nicolai, Fabio Girardi, Cesare Piazza

**Affiliations:** 1 Section of Otorhinolaryngology, Head and Neck Surgery, ASST Spedali Civili Di Brescia, University of Brescia, Piazza Spedali Civili 1, 25123 Brescia, Italy; 2 Department of Medical and Surgical Specialties, Radiological Sciences and Public Health, University of Brescia, Brescia, Italy; 3 Section of Medical Oncology, ASST Spedali Civili Di Brescia, University of Brescia, Brescia, Italy; 4 Section of Pathology, ASST Spedali Civili Di Brescia, University of Brescia, Brescia, Italy; 5 Section of Otorhinolaryngology, Head and Neck Surgery, Azienda Ospedaliera Di Padova, University of Padua, Padua, Italy; 6 Integrated Oncology Center of Ana Nery Hospital, Santa Cruz Do Sul, RS Brazil

**Keywords:** Squamous cell carcinoma, Skin cancer, Lymph node metastasis, Prognosis, Parotid

## Abstract

**Purpose:**

To identify potential risk factors impacting on overall survival (OS) of patients affected by lymph node metastasis from cutaneous squamous cell carcinoma (cSCC) of the head and neck (HN), with special emphasis on primary tumor characteristics and pattern of nodal recurrence (intraparotid and/or cervical).

**Methods:**

A bi-institutional retrospective study on consecutive patients affected by cervical and/or intraparotid NM from HN cSCC and surgically treated with curative intent from May 2010 to January 2020 was conducted. OS was considered the outcome of interest.

**Results:**

The study included 89 patients (M:F = 3.4:1; median age, 78 years; range, 22–99). Among the primary tumor characteristics, the most relevant prognostic factors were diameter ≥ 4 cm (hazard ratio [HR] = 2.56, *p* = 0.010) and depth of infiltration ≥ 6 mm (HR = 3.54, *p* = 0.027). Cervical NM was associated with worse OS (HR = 2.09, *p* = 0.016) compared to purely intraparotid NM (5-year OS: 60.9% vs. 28.1%, *p* = 0.014). At multivariable analysis, age, immunosuppression, pT3-T4 categories and a high burden of nodal disease (> 2 NM) confirmed to be independent risk factors, whereas adjuvant radiotherapy was independently associated with better outcome.

**Conclusion:**

This study confirms the association of several independent prognosticators related to the patient, primary tumor, and nodal burden status. Patients with cervical NM should be considered at risk for harboring a higher number of metastatic lymph nodes.

## Introduction

Non-melanoma skin cancer is one of the most common malignancies worldwide, and the head and neck (HN) region is among the most frequently affected sites due to sun exposure. Whereas the vast majority of these tumors is represented by basal cell carcinoma (70–80% of cases), cutaneous squamous cell carcinoma (cSCC) accounts for nearly 20% of cases. In the United States, roughly 200,000–400,000 new cases of cSCC are diagnosed yearly. Of those, approximately 40,000 cases present at advanced stages with an estimated 15,000 deaths each year [1]. A study from Nasser in 2011 found an incidence rate of cSCC in the Brazilian population of 120 cases/100,000 inhabitants, reaching a peak of 1484 cases/100,000 in males and 975 cases/100,000 in females aged 70 years or more [2]. The etiology is heterogeneous: sun exposure is recognized to be the primary cause, followed by additional risk factors such as genetic predisposition, previous skin lesions, immunosuppression (IS), and chronic trauma [3, 4].

Survival of patients affected by cSCC is mostly influenced by the development of lymph nodes metastases (NM), while distant spread is quite rare [5, 6]. NM occurs in fewer than 5% of patients, although this estimation may be biased by the absence of prospective tumor registries and the multi-specialistic management of these patients [7–9]. Well-known risk factors for NM are advanced tumor category, presence of perineural (PNI) or lympho-vascular invasion (LVI), recurrent disease, and previous transplant or other causes of IS [1]. Compared to SCC occurring in the upper aerodigestive tract [10, 11], survival of regional metastatic cSCC is higher, with 5-year estimates between 50 and 70% [12, 13].

An essential tool for physicians managing this specific subset of patients is represented by the American Joint Committee on Cancer (AJCC)—Union for International Cancer Control (UICC) TNM staging system [14]. This incorporates substantial features affecting cSCC prognosis on an evidence-based level. Starting from the 7th Edition [15], in fact, important elements were included such as depth of invasion (DOI), PNI, LVI, and grading. Subsequently, in the 8th Edition, other pivotal features have been incorporated: primary tumor diameter ≥ 4 cm, subdermal neural invasion, bone erosion, DOI ≥ 6 mm, and subdermal plane involvement. The presence of extranodal extension (ENE) was also included in the cSCC staging system, although only one study so far has shown an association between this prognosticator and survival in patients with NM non-concomitant with the primary lesion [16–18].

While the risk profile of primary non-metastatic HN cSCC has been well described in several published studies [19–21], thorough data for patients with NM from HN cSCC are still lacking. Few papers have specifically analyzed the subgroup of cSCC with NM [22–24]*,* but primary tumor characteristics were not addressed [22], or study was limited to patients with intraparotid metastasis [23, 24] The primary objective of this study was to retrospectively analyze a cohort of patients affected by HN cSCC with intraparotid and/or cervical NM, and to clarify how primary tumor characteristics and pattern of regional disease presentation affect overall survival (OS).

## Materials and methods

A retrospective study on patients affected by neck and/or intraparotid NM from cSCC of the HN region was conducted at the Departments of Otorhinolaryngology—Head and Neck Surgery of the University of Brescia, Italy, and at the Integrated Oncology Center of Ana Nery Hospital, Santa Cruz do Sul, Brazil. All consecutive patients surgically treated from May 2010 to January 2020 at both institutions were identified in electronic medical records using the 10th revision of the International Classification of Diseases (ICD-10) codes (C07/C77.0 and C44) and selected for the study. Moreover, all pathological reports were retrospectively reviewed by searching for parotidectomy and/or neck dissection and previous or concomitant history of HN cSCC. Exclusion criteria were age < 18 years, presence of distant metastasis at diagnosis, and surgery performed with palliative intent. In the majority of cases patients were referred to our Units for surgical treatment of clinically evident NM, while primary tumors were mostly treated in other units of the same Italian and Brazilian institutions (such as dermatology or plastic surgery departments). Accordingly, a retrospective collection of data on primary cutaneous tumor characteristics (tumor largest diameter, DOI, differentiation, presence of PNI, LVI, and resection margins) was achieved consulting electronic pathologic reports on local databases. In case of metachronous ipsilateral multiple HN cSCC resections, if clinical information could not definitively settle the primary identification, the last one removed was considered the metastasizing lesion.

Patients were treated in accordance with the National Comprehensive Cancer Network Guidelines for HN cSCC [25]; specifically, adjuvant chemo-radiotherapy (CRT) was performed in case of ENE + , and/or more than 2 positive nodes, whenever feasible according to the performance status of the patient. The most relevant clinical-pathological features (demographic and clinical data, pathologic details of the primary tumor, number of NM and location within the parotid gland and/or neck, disease staging, details of surgery for primary tumor and NM, and adjuvant treatment) were retrieved (Tables 1, 2). All tumors were classified according to the TNM 8th Edition and O’Brien classification (Table 3) [26]. The study was conducted in accordance with the Declaration of Helsinki, approved by the local ethic committees at both centers (Ref CAAE: 93792318.4.0000.5304 and NP4266), and data were anonymized.

**Table 1:** Descriptive statistics showing characteristics of patients and treatment for nodal disease

Variable	No.	%
Patient characteristics		
Gender		
Male	69	77.5
Female	20	22.5
Age (years)		
Median	78	
Range	22–99	
Immunosuppression		
Absent	77	86.5
Present	12	13.5
Treatment of nodal metastasis		
INT T-N (months)		
Median Range	8 0–88	
Type of surgery		
Parotidectomy + ND	63	70.8
Parotidectomy (exclusive)	13	14.6
ND (exclusive)	13	14.6
Type of parotidectomy (*N* = 76)		
Superficial—subtotal	26	34.2
Total	34	44.7
Radical	15	19.7
Non specified	1	1.3
Type of neck dissection (*N* = 76)		
SND	47	61.8
MRND	26	34.2
RND	3	3.9
Adjuvant treatment		
None	36	40.4
RT	47	52.8
CRT	6	6.7

*CRT* chemoradiotherapy, *INT T-N* interval between primary tumor and nodal occurrence, *ND* neck dissection, *MRND* modified radical neck dissection, *RND* radical neck dissection, *RT* radiotherapy, *SND* selective neck dissection

**Table 2:** Descriptive statistics of the most relevant characteristics of primary tumor and nodal metastasis

Variable	No.	%
Primary tumor characteristics		
Subsite		
Cervical	4	4.5
Auricle	25	28.1
Fronto-temporal	22	24.7
Lower lip	5	5.6
Mandibular region	3	3.4
Malar region	14	15.7
Nose	7	7.9
Vertex	9	10.1
Largest diameter (mm)		
Median	25	
Range	8–55	
Largest diameter		
< 4 cm	62	69.7
≥ 4 cm	17	19.1
Missing	10	
DOI (mm)		
Median	8.30	
Range	1.5–50	
DOI (mm)		
≤ 6 mm	43	61.4
> 6 mm	27	38.6
Missing	19	
Primary tumor differentiation		
Well differentiated (G1)	17	20.7
Moderately differentiated (G2)	38	46.4
Poorly differentiated (G3)	27	32.9
Missing	7	
PNI		
Absent	60	67.4
Present	29	32.6
LVI		
Absent	59	66.3
Present	30	33.7
Margin status		
R0	65	75.6
R1	21	24.4
Missing	3	
pT classification		
T1	21	25.3
T2	27	32.5
T3	33	39.8
T4	2	2.4
Missing	6	
Nodal disease characteristics		
Overall number of nodal metastasis		
Median	2	
Range	1–10	
Extranodal extension (ENE)		
Absent	11	12.8
Present	75	87.2
Missing	3	
Location of nodal metastases		
Parotid	42	47.2
Cervical	21	23.6
Both	26	29.2
Exclusive intraparotid nodal metastasis		
Median number	1	
Range	1–5	
ENE−	6	15.0
ENE +	34	85.0
Missing	2	
Exclusive cervical nodal metastasis		
Median number	2	
Range	1–9	
ENE−	4	20.0
ENE +	16	80.0
Missing	1	
Nodal metastasis to both parotid and neck		
Median number	4	
Range	2–10	
ENE−	1	3.8
ENE +	25	96.2
pN classification		
Exclusive parotid N +	26	29.2
pN1	4	4.5
pN2	11	12.4
pN3	48	53.9
O’Brien classification		
P1N0	14	15.7
P0N1	6	6.7
P0N2	14	15.7
P1N2	4	4.5
P2N0	19	21.3
P2N2	3	3.4
P3N0	12	13.5
P3N1	5	5.6
P3N2	3	3.4

*DOI* depth of infiltration, *LVI* lympho-vascular invasion, *PNI* perineural invasion

**Table 3:** O’Brien classification system of intraparotid and neck nodal metastases

Parotid	
P0	No clinical disease in the parotid
P1	Metastatic node up to 3 cm diameter
P2	Metastatic node more than 3 cm up to 6 cm diameter or multiple parotid nodes
P3	Metastatic node more than 6 cm in diameter or disease involving VII nerve or skull base
Neck	
N0	No clinical disease in the neck
N1	Single ipsilateral neck node up to 3 cm diameter
N2	Single node more than 3 cm diameter or multiple neck nodes or contralateral nodes

### Statistical analysis

Variables were expressed in terms of median, range of values, and percentages. OS was considered as the primary outcome; time to death and the most recent clinical-radiological information (censored observations) were evaluated. Demographics, primary tumor, NM characteristics, and treatment-related variables were considered.

Univariate OS analyses were conducted with the Cox proportional hazard model and log-rank test. Results were expressed in terms of hazard ratio (HR) and 5-year OS estimates, respectively, with relative 95% confidence intervals (CI). The Kaplan–Meier method was used to graphically represent the OS of the entire cohort and according to the most significant variables affecting OS with the relative 95% CI and the table of number of patients at risk by time. Survival curves were plotted up to the maximum available follow-up, to better represent long-term survival.

A multivariable Cox proportional hazard model was carried out considering relevant prognostic factors known from the literature, excluding multi-collinearity between covariates according to a variance inflation factors (vif) < 5. Martingale and Schoenfeld residuals were evaluated for the assessment of linear effect for continuous variables and proportional hazards assumptions.

Statistical analysis was performed using R (version 4.0.3, R Foundation for Statistical Computing, Vienna, Austria). *p* values < 0.05 were considered statistically significant.

## Results

### Patient’s characteristics

The study included 89 patients with cervical and/or intraparotid NM from HN cSCC (Table 1). There were 69 males and 20 females (M:F = 3.4:1). Median age at diagnosis was 78 years (range 22–99). IS was documented in 12 (13.5%) patients and was always secondary to organ transplantation. Concurrent surgery was performed on the T and N sites in 11 (12.3%) patients, while 78 (87.6%) received surgery on NM after treatment of the primary (with a median interval of 8 months; range 1–48). Patients treated in other departments for primary HN cSCC accounted for 52.8% of cases. Median follow-up was 11 months (range 1–111 months). Characteristics of the primary tumor are summarized in Table 2. We were able to collect specific data regarding the largest diameter in 87.6%, DOI in 55.1%, tumor differentiation in 92.1%, and presence of PNI, LVI and margin status in 96.6% of cases. Median diameter was 25 mm, while median DOI was 8.3 mm. The most frequently involved subsites were: auricle (*n* = 25, 28.1%), fronto-temporal region (*n* = 22, 24.7%), and malar area (*n* = 14, 15.7%). Most of the primary tumors (53.9%) were classified as T1–T2, while high-grade features (G3) were observed in 32.9%, PNI in 32.6%, and LVI in 33.7% of cases. Resection margins were negative in 65 (75.6%) patients, and positive or close in 21 (24.4%). The deep margin was the most frequently involved at pathologic evaluation (*n* = 13, 15.1%).

Details on treatment for NM are listed in Table 1. Most patients (70.8%) underwent both parotidectomy and neck dissection, whereas the remaining cases were equally treated by exclusive parotidectomy or neck dissection alone (14.6% each). Total, superficial, and radical parotidectomy were performed in 44.7%, 34.2%, and 19.7% of patients, respectively. Selective neck dissection was the most common intervention on the neck (61.8%), followed by modified radical (34.2%), and radical neck dissections (3.9%). At histopathology (Table 2), NM involving both intraparotid and cervical lymph nodes were observed in 29.2% of cases, whereas exclusive intraparotid or cervical metastases were found in 47.2% and 23.6% of cases, respectively. The overall number of NM ranged from 1 to 10 (median, 2), and ENE was observed in 87.2% of cases When exclusive intraparotid and cervical nodes were involved, the median number of NM were 1 and 2, respectively, whereas ENE was observed in 85% and 80% of cases, respectively. When concomitant intraparotid and neck nodes were involved, the median number of NM increased to 4, with ENE observed in 96.2% of cases.

In summary, 29.2% of patients were affected by exclusive intraparotid NM. Neck metastases were observed in 71.8% of patients: 6.3% were classified as pN1, 17.5% as pN2, and 76.2% as pN3. Considering the O’Brien classification (Table 3), most lesions were classified as stage P2N0 (21.3%), followed by P0N2 (15.7%), P1N0 (15.7%), and P3N0 (13.5%). All the other O’Brien stages had a frequency < 10%.

Postoperative adjuvant therapy was administered in 61.6% of patients: 88.7% received exclusive radiotherapy (RT) and 11.3% combined CRT. A total of 3 patients previously received adjuvant RT, one on the primary site (total dose, 60 Gy), one on the ipsilateral neck from level I to IV (total dose, 54 Gy), and one on both sides of the neck due to regional recurrence secondary to a lower lip SCC (staged as rypN2a).

### Overall survival and analysis of prognosticators

**Fig. 1:**
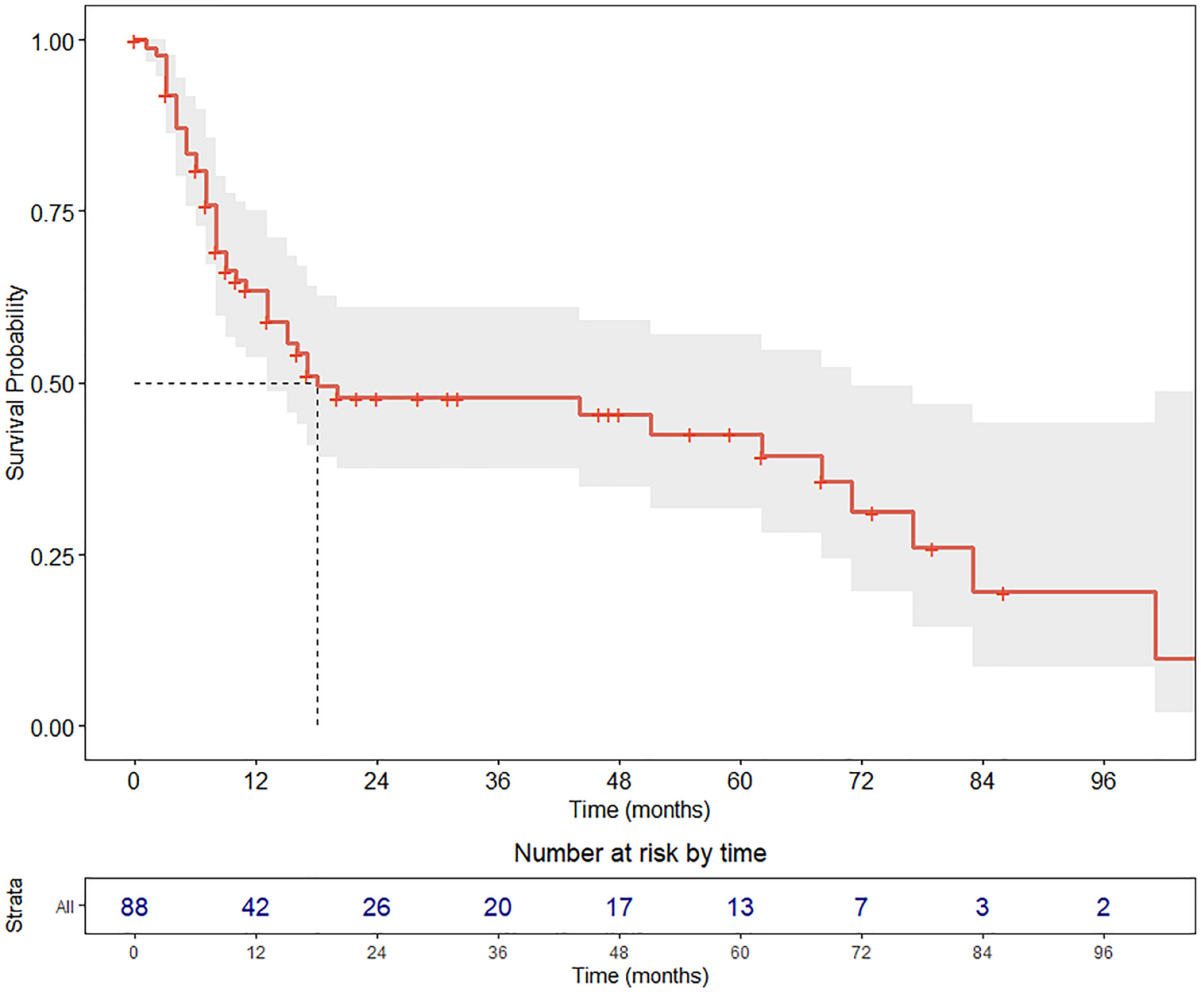
Kaplan Meier survival curve showing OS in the cohort of nodal metastatic patients. Median survival is depicted with the dashed line

The 2- and 5-year OS were 47.8% (95% CI, 37.4–61.0%) and 42.6% (95% CI, 31.8–57.1%), respectively (Fig. 1). At univariate analysis (Table 4), age was a significant risk factor for OS with a linear effect (HR = 1.04, *p* = 0.010). On the contrary, gender and IS did not show a significant impact on 5-year OS. The interval between primary tumor and NM treatment (INT T-N) seems to not represent a risk factor (*p* = 0.386).

**Table 4:** Univariate and multivariable analysis

Overall Survival (OS)		Univariate analysis				Multivariable analysis	
		Log-rank test		Cox proportional hazard regression model		Cox proportional hazard regression model	
Variables		5-yr OS (95% CI)	*p* value	HR (95% CI)	*p* value	HR (95% CI)	*p* value
Age–years				1.04 (1.01–1.06)	**0.010**	1.04 (1.00–1.07)	**0.028**
Gender	Female	45.3% (26.4–77.8)	0.879	REF			
	Male	41.7% (29.6–58.9)		1.05 (0.52–2.13)	0.880		
Immunosuppression	Absent	45.5% (34.2–60.6)	0.138	REF		REF	
	Present	23.1% (5.04–100)		1.80 (0.83–3.88)	0.137	3.15 (1.19–8.31)	**0.020**
INT T-N—months				1.01 (0.98–1.04)	0.386	1.04 (0.99–1.08)	*0.055*
Major diameter T	≤ 40 mm	50.3% (37.4–67.7)	**0.008**	REF			
	> 40 mm	24.4% (8.0–74.1)		2.56 (1.25–5.23)	**0.010**		
DOI T	≤ 6 mm	66.7% (40–100)	**0.019**	REF			
	> 6 mm	31.2% (16–61)		3.54 (1.16–10.87)	**0.027**		
Primary tumor differentiation	Well-moderately differentiated (G1–G2)	46.0% (33.6–62.9)	0.946	REF			
	Poorly differentiated (G3)	31.9% (14.2–71.5)		1.02 (0.52–2.00)	0.958		
PNI (primary tumor)	Pn0	52.8% (39.8–70.2)	**0.047**	REF			
	Pn1	26.0% (11.6–57.9)		1.86 (1.00–3.46)	**0.050**		
LVI (primary tumor)	Lv0	50.9% (37.5–69.1)	0.156	REF			
	Lv1	30.4% (15.5–59.6)		1.54 (0.85–2.81)	0.158		
Margins	R0	47.2% (34.6–64.5)	*0.094*	REF			
	R1	30.8% (13.7–68.9)		1.74 (0.89–3.38)	0.104		
pT stage	T1–2	53.7% (37.9–74.1)	**0.004**	REF			
	T3–4	27.3% (14.4–51.9)		2.34 (1.27–4.34)	**0.005**	4.53 (2.09–9.80)	** < 0.001**
Overall number of nodal metastasis	≤ 2	52.6% (39.3–70.5)	**0.014**	REF			
	> 2	22.8% (10.0–52.0)		2.04 (1.14–3.67)	**0.017**	2.36 (1.04–5.36)	**0.040**
ENE	ENE−	24.2% (7.4–79.2)	0.600	REF			
	ENE +	44.4% (32.4–61)		0.80 (0.35–1.81)	0.596	0.52 (0.20–1.33)	0.171
Distribution of nodal metastasis	Exclusive parotid	60.9% (45.5–81.7)	**0.047**	REF			
	Exclusive neck	25.7% (11.7–56.6)		2.03 (1.01–4.08)	**0.047**	1.63 (0.67–4.00)	0.283
	Parotid and neck	33.3% (17.7–62.6)		2.18 (1.07–4.43)	**0.033**	1.49 (0.51–4.38)	0.470
Presence of neck metastasis	Absent	60.9% (45.5–81.7)	**0.014**	REF			
	Present	28.1% (16.3–48.5)		2.09 (1.15–3.82)	**0.016**		
pN stage	Exclusive parotid pN +	60.6% (41.8–87.9)	0.62	REF			
	pN1	33.3% (67.3–100)		0.80 (0.17–3.72)	0.780		
	pN2	51.9% (26.6–100)		1.03 (0.35–3.04)	0.963		
	pN3	34.8% (21.8–55.5)		1.46 (0.71–2.99)	0.303		
O’Brien classification*	P1N0	76.6% (56.5–100)	**0.009**	REF			
	P0N1	66.7% (37.9–100)		1.15 (0.27–4.82)	0.850		
	P0N2	15.7% (4.2–55.9)		4.14 (1.44–11.94)	**0.009**		
	P1N2	21.7% (4.6–100)		6.17 (1.45–26.3)	**0.013**		
	P2N0	57.2% (37.1–88.1)		1.62 (0.55–4.77)	0.380		
	P2N2	66.7% (3–100)		2.37 (0.46–12.24)	0.305		
	P3N0	71.4% (44.7–100)		1.20 (0.23–6.27)	0.828		
	P3N1	33.3% (6.7–100)		2.01 (0.39–10.47)	0.407		
	P3N2	0%		7.98 (1.84–34.60)	**0.006**		
Adjuvant RT	No	26.6% (14.7–48.2)	** < 0.001**	REF		REF	
	Yes	54.8% (40.1–74.9)		0.29 (0.16–0.56)	**0.001**	0.29 (0.12–0.70)	**0.006**

Bold type represents statistically significant values

*CI* confidence interval, *INT T–N* interval between primary tumor and nodal occurrence, *DOI* depth of infiltration, *ENE* extranodal extension, *HR* hazard ratio, *LVI* lympho-vascular invasion, *OS* overall survival, *PNI* perineural invasion, *REF* reference value (HR = 1), *RT* radiotherapy

^*^Survival data for O’Brien classification are referred to 2-yr OS

**Fig. 2:**
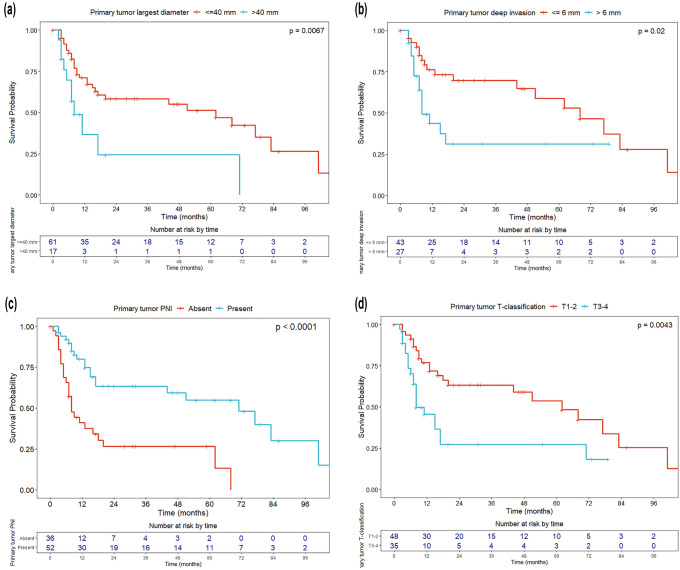
Overall survival according to primary tumor most relevant characteristics: **a** primary tumor largest diameter according to the cut-off of 4 cm, **b** primary tumor depth of infiltration according to the cut-off of 6 mm, **c** absence or presence of primary tumor perineural invasion (PNI), and **d** pT category according to the 8^th^ Edition of the AJCC-UICC TNM classification (12)

Among the characteristics of the primary tumor, the most relevant prognosticators in terms of OS were the largest diameter (> 4 cm, HR = 2.56, *p* = 0.010) and DOI (> 6 mm, HR = 3.54, *p* = 0.027). Consequently, pT3-T4 primary lesions showed a significant decrease in 5-year OS (27.3% vs. 53.0%, *p* = 0.007; HR = 2.34, *p* = 0.006). The presence of PNI was significantly associated with worse OS (HR = 1.85, *p* = 0.05), whereas the site of origin, tumor differentiation, resection margins, and LVI did not reach statistical significance (Fig. 2).

**Fig. 3:**
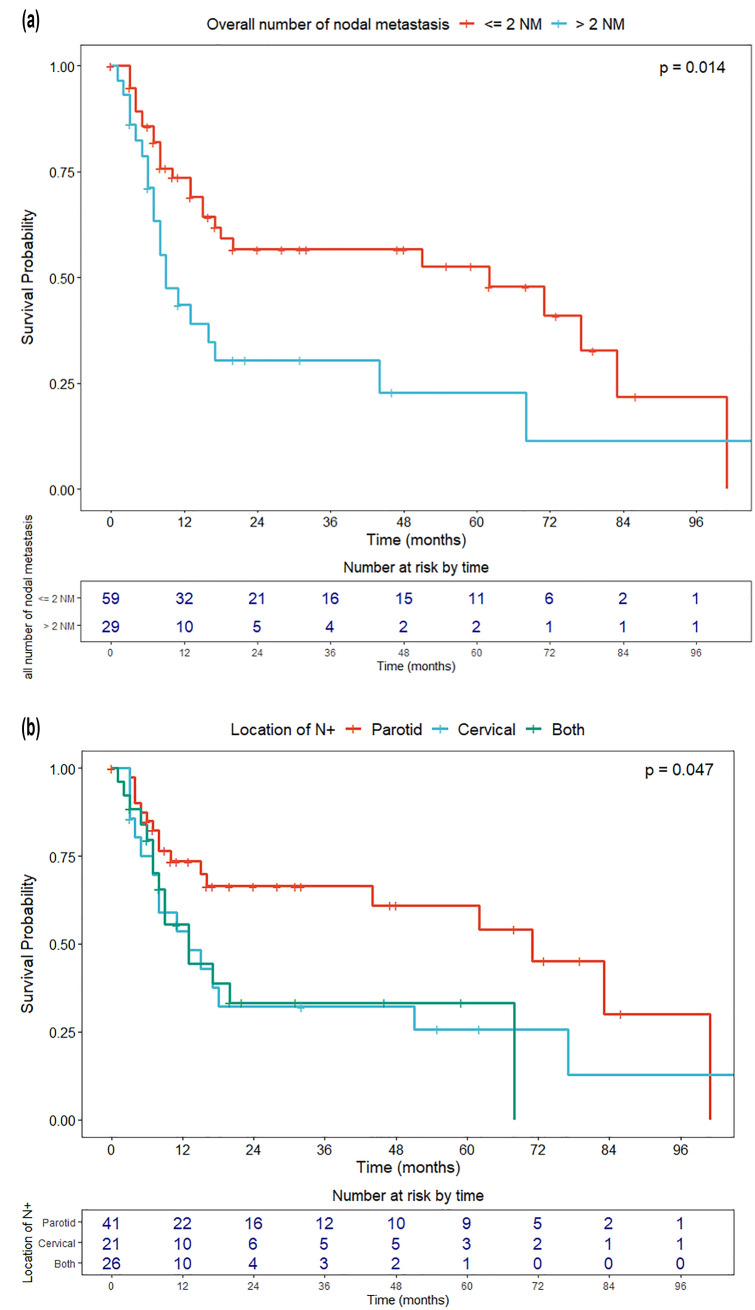
Overall survival according to **a** burden of nodal disease (low, ≤ 2 NM and high, > 2 NM), and **b** localization of regional metastasis (exclusive intraparotid, exclusive cervical, and both intraparotid and cervical)

**Fig. 4:**
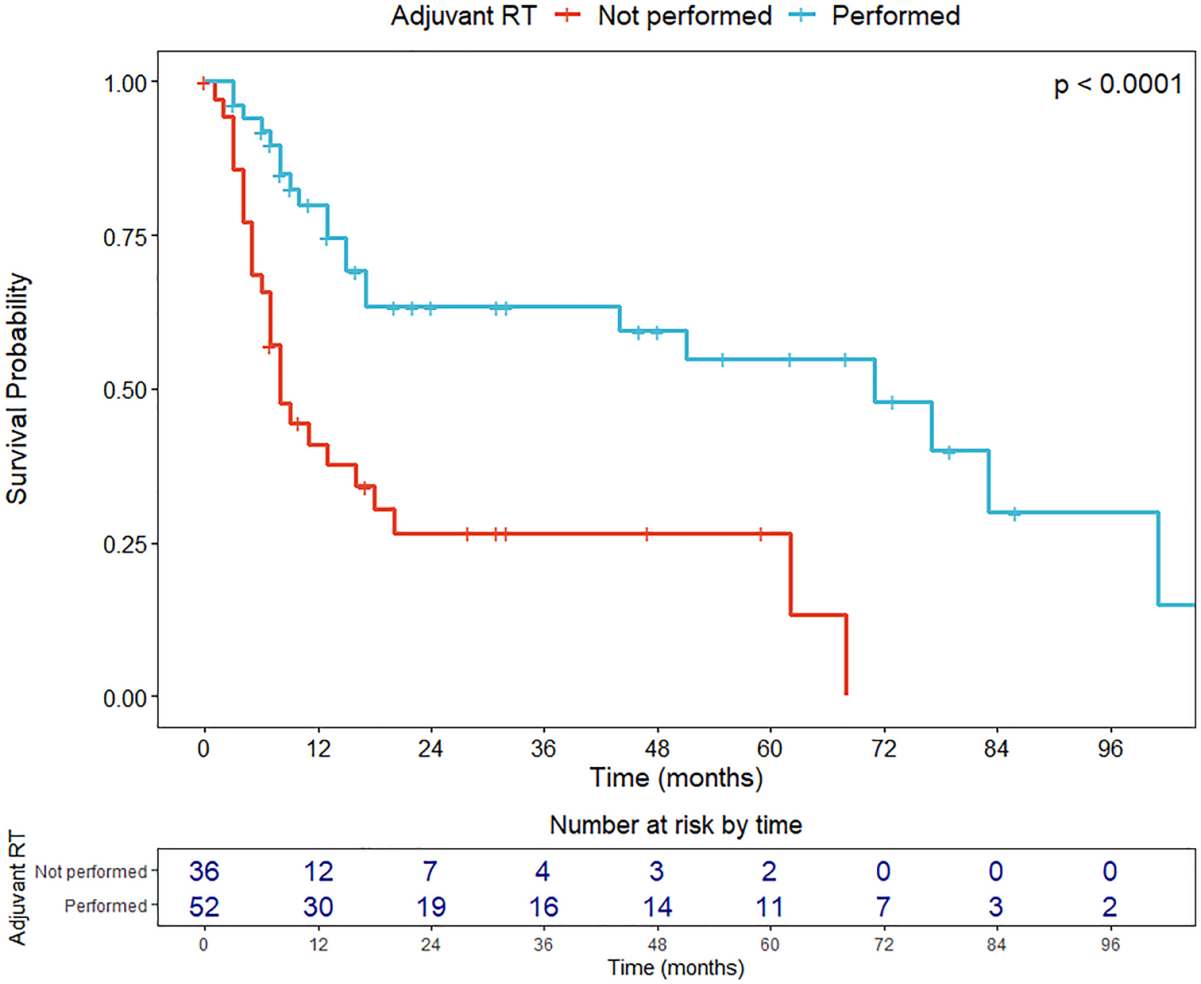
Role of adjuvant radiotherapy, with/without concurrent chemotherapy, in relation to overall survival

Regarding the parameters related to regional disease, the overall number of NM, categorized according to the median value, was a significant negative prognosticator (NM > 2, HR = 2.04, *p* = 0.017) (Fig. 3a). At the same time, distribution of NM affected OS, since cervical metastasis (exclusive or concomitant with intraparotid localizations) were associated with a significant increase in mortality compared to exclusive intraparotid nodal involvement (overall, HR = 2.09, *p* = 0.016; exclusive cervical, HR = 2.03, *p* = 0.047; concomitant cervical and intraparotid nodes, HR = 2.18, *p* = 0.033, respectively) (Fig. 3b). This finding was confirmed by analysis of the O’Brien classification [26]. When compared to an exclusive low burden of parotid disease (P1N0), only patients with a high burden of cervical metastasis (N2) showed a significant decrease in OS. Conversely, ENE (*p* = 0.600), type of parotidectomy performed (superficial vs total vs radical, *p* = 0.995), and pN staging according to the 8th Edition (*p* = 0.620) did not influence OS. Finally, adjuvant RT was a relevant protective factor (HR = 0.29, *p* 0.010) (Fig. 4).

At multivariable analysis (Table 4), age (HR = 1.04, *p* = 0.028), IS (HR = 3.15, *p* = 0.020), pT3-T4 categories (HR = 4.53, *p* < 0.001), high burden of nodal disease (NM > 2, HR = 2.36, *p* = 0.040), and adjuvant RT (HR = 0.42, *p* = 0.053) were independent prognosticators.

## Discussion

The main observation provided by our study is that age, superficial tumor diameter, DOI, pT category, number/site of NMs, and postoperative RT have a significant impact on OS of patients affected by HN cSCC. Overall, these findings are in line with a recent meta-analysis that pooled 3534 patients affected by regional metastatic HN cSCC [27]. The authors stated that risk factors significantly impacting on OS were: IS (HR = 2.66; 95% CI, 2.26–3.13), ENE (HR = 1.90; 95% CI, 1.12–3.23), adjuvant RT (HR = 0.45; 95% CI, 0.27–0.78), high lymph node ratio (HR = 1.91; 95% CI, 1.09–3.35), and advanced age (HR = 1.03; 95% CI, 1.00–1.07) [27]. Moreover, we further demonstrated that number of NM greater than 2 is an independent negative prognosticator that overwhelms the weight of ENE and nodal site.

In our study, 2- and 5-year OS estimates were 47.8% and 42.6%, respectively, which are slightly lower than those presented in other published series [12, 13]. Of note, the median age in our cohort was quite advanced (78 years), and most patients presented with ENE. Age was a significant negative prognosticator for OS at both univariate (*p* < 0.01) and multivariable analysis (*p* < 0.05). This finding has also been recently confirmed by Bobin et al. [23] in a study on 35 patients (with a mean age of 76.3 years) affected by NM within the parotid gland. The authors found that age impacts only on OS, but not on disease specific survival (DSS). This underlines the utmost importance of correct pre-treatment comprehensive geriatric assessment of the elderly and frail prior to defining the most appropriate therapeutic journey [28].

IS definitively plays a pivotal role in the development of advanced primary and metastatic cSCC, being associated with a decreased OS and DSS [29]. In a publication by Euvrard et al. [30], important issues are raised regarding the nature of IS itself (for example, use of immunosuppressant medications is considered worse compared to infection from human immunodeficiency virus and even AIDS), and persistence vs. reversibility of the IS condition. McDowell et al., in a series of 132 HN cSCC with intraparotid NMs, found that IS was the main prognosticator, with 14% 5-year OS vs. 53% in non-IS patients [31]. Moreover, Martinez et al. analyzed the data from 68 organ transplant recipients with 73 distinct metastatic skin cancers finding that, at 1 year after appearance of metastasis, the cumulative incidence of relapse was 29% and the 3-year DSS was 56%. The authors concluded that, in this specific subset of patients, the prognosis is poor and the chance of developing more aggressive disease with NM is higher compared to immunocompetent patients [32]. In our cohort, IS was present in 13.4% of cases, all for drug-related permanent causes after organ transplantation. Although not statistically significant at univariate analysis, patients with IS experienced a relevant decrease in survival (5-year OS 23.1% vs. 45.5%). Moreover, IS was an independent risk factor in multivariable analysis, with a relevant HR. This observation, which is consistent with the results of a recent meta-analysis, highlights the importance of considering IS in defining the risk profile of patients with cSCC, and identifies a possible limitation in the current staging system of these tumors [33].

Many pathologic features of primary tumor, namely its largest diameter, DOI, and PNI, proved to be significant prognosticators in terms of OS in metastatic patients, thus suggesting the importance of considering these characteristics when dealing with treatment of NM.

In the 8th Edition of the UICC-AJCC Cancer Staging Manual [14], important changes regarding the definition of locally advanced disease (T3–T4) were introduced: superficial diameter > 4 cm, DOI > 6 mm, infiltration beyond the subdermal fat, and gross neural involvement. Our analysis validated the prognostic cut-offs introduced for the largest diameter and DOI in terms of OS also in a cohort of patients with NM. Furthermore, locally advanced primary lesion (pT3-T4) proved to be an independent prognosticator in a metastatic setting at multivariable analysis.

High burden of nodal disease (> 2 NM) was an independent risk factor, significantly affecting OS. Our findings are in line with the previous experience of Ebrahimi et al. showing that the increasing number of NM (categorized as 1–2, 3–4, and > 5) is an independent predictor of mortality [34]. Notably, we confirmed the cut-off set at > 2 NM as appropriate in discriminating between high and low burden of nodal disease.

Regarding the pattern of regional disease, presence of neck NM was associated with a relevant decrease in OS compared to exclusive parotid involvement (Fig. 3) at the univariate analysis. This finding may be explained by the higher number of NM found when cervical nodes were involved.

In 2002, O’Brien et al. introduced a classification for regional metastases of cSCC and compared the prognostic impact of intraparotid and neck NM. The authors demonstrated that an increase of *P* stage is directly correlated with a reduced local disease control, but not with a decrease in OS, while patients with a high neck nodes burden show an independent reduction in OS [26]. Interestingly, the AJCC-UICC 8th Edition N classification [14] failed to reach statistical significance in our analysis. Overall, these findings may call for a revision of the N classification of these tumors, which should primarily consider the number of positive nodes as a major criteria for prognostic stratification. Although ENE is recognized to be a major prognosticator [27], it was not in our analysis. This finding should be interpreted cautiously and could be related to the small number of ENE-negative patients (12.4%). The remarkable incidence of ENE in our series may be explained by the advanced age of the population but could also be the result of NM delayed diagnosis. Treatment delay could have multiple causes: lack of radiological staging of nodal area at first diagnosis, absence of an evaluation of the locally advanced cases in a multidisciplinary setting, and incorrect planning or adherence to the follow-up schedule.

Finally, RT was delivered in about half of patients and turned out to be an independent protective factor. This finding was also confirmed in previous papers [35–38]. Larger prospective studies to better define the benefit of RT and place it in relation to nodal burden and pN category are definitely warranted.

The present study has some limitations. Despite the inclusion of metastatic patients only, the sample size is still relatively small, which can reduce the statistical power of the analysis. Second, the retrospective design is limited by possible selection bias and reduced quality of data, although minimized by a meticulous chart review. Finally, a large portion of the cohort was surgically treated on the primary tumor at other units/centers. This can represent a bias due to inadequate primary tumor treatment or improper follow-up, which can lead to a higher rate of loco-regional recurrence or a delayed treatment of NM than what is expected in large-volume tertiary care centers.

## Conclusion

Advanced T category, high burden of nodal metastasis (> 2 NM), IS, and age emerged as major independent negative prognosticators, while adjuvant RT showed a relevant protective role. These data, combined with other well-established prognosticators, can help in identifying a subgroup of cSCC that may require intensified treatments and closer follow-up. Moreover, identification of specific patient and disease characteristics may help in recruiting for clinical trials with adjuvant immunotherapy, which is one of the most promising strategies for cSCC.
